# Protective effect of geranylgeranylacetone, an inducer of heat shock protein 70, against drug-induced lung injury/fibrosis in an animal model

**DOI:** 10.1186/1471-2466-9-45

**Published:** 2009-09-16

**Authors:** Takayoshi Fujibayashi, Naozumi Hashimoto, Mayumi Jijiwa, Yoshinori Hasegawa, Toshihisa Kojima, Naoki Ishiguro

**Affiliations:** 1 Department of Orthopedic Surgery, Nagoya University Graduate School of Medicine, Nagoya, Japan; 2 Department of Respiratory Medicine, Nagoya University Graduate School of Medicine, Nagoya, Japan; 3 Department of Pathology, Nagoya University Graduate School of Medicine, Nagoya, Japan

## Abstract

**Background:**

To determine whether oral administration of geranylgeranylacetone (GGA), a nontoxic anti-ulcer drug that is an inducer of heat shock protein (HSP) 70, protects against drug-induced lung injury/fibrosis *in vivo*.

**Methods:**

We used a bleomycin (BLM)-induced lung fibrosis model in which mice were treated with oral 600 mg/kg of GGA before and after BLM administration. Inflammation and fibrosis were evaluated by histological scoring, hydroxyproline content in the lung and inflammatory cell count, and quantification by ELISA of macrophage inflammatory protein-2 (MIP-2) in bronchoalveolar lavage fluid. Apoptosis was evaluated by the TUNEL method. The induction of HSP70 in the lung was examined with western blot analysis and its localization was determined by immunohistochemistry.

**Results:**

We confirmed the presence of inflammation and fibrosis in the BLM-induced lung injury model and induction of HSP70 by oral administration of GGA. GGA prevented apoptosis of cellular constituents of lung tissue, such as epithelial cells, most likely related to the *de novo *induction of HSP70 in the lungs. GGA-treated mice also showed less fibrosis of the lungs, associated with the findings of suppression of both production of MIP-2 and inflammatory cell accumulation in the injured lung, compared with vehicle-treated mice.

**Conclusion:**

GGA had a protective effect on drug-induced lung injury/fibrosis. Disease-modifying antirheumatic drugs such as methotrexate, which are indispensable for the treatment of rheumatoid arthritis, often cause interstitial lung diseases, an adverse event that currently cannot be prevented. Clinical use of GGA for drug-induced pulmonary fibrosis might be considered in the future.

## Background

Rheumatoid arthritis (RA), a chronic, systemic, inflammatory autoimmune disease, causes irreversible joint deformities and functional impairment. Evidence accumulating over the past 10 years has suggested that combined treatment with disease-modifying antirheumatic drugs (DMARDs), especially methotrexate, and anti-TNFα biological agents, as early as possible after the diagnosis of RA is effective and critical for preventing substantial disability caused by bony erosions [1-4]. On the other hand, it is also well known that DMARDs and other drugs for RA have various adverse effects, which require discontinuation or revision of the therapeutic schedule [5,6]. Since drug-induced interstitial pneumonitis is a well-known complication among these adverse effects that is often life-threatening [7-9], investigation for preventing its onset during the treatment for RA is warranted. Although the mechanism by which DMARDs cause interstitial pneumonitis remains unclear, clinical and histopathological evaluations suggest that it shares many features with idiopathic interstitial pneumonitis (IIP) [10,11], allowing us to speculate that the underlying mechanisms in the pathogenesis of IIP might be also involved in those in drug-induced interstitial pneumonia.

Although the pathological roles of cell injury and apoptosis in the epithelium of the airways during acute lung injury and fibrosis have not been fully determined, increased apoptosis of alveolar epithelial cells (AECs) has been observed in IIP, not only in the fibrotic lesions, but also in histologically normal alveoli [12-15].

The heat-shock proteins (HSPs) have a cytoprotective property as intracellular chaperones, by which aberrantly folded or mutated proteins involved in various stressful conditions are repaired and, if necessary, degraded for cellular homeostasis [16,17]. A wide variety of stresses, such as ischemia and inflammation, induce an increase in the expression of HSPs, and HSP70 in particular has been proved to not only exhibit a cytoprotective function through anti-apoptosis processes against stress *in vitro*, but to also exert strong cytoprotection in the stomach, liver, and heart *in vivo *[18-21].

Geranylgeranylacetone (GGA), a nontoxic anti-ulcer drug, has been recently shown to induce HSPs, especially HSP70, in various animal disease models, which suggests that the administration of GGA would have a protective effect against several injury models and human disease [22-24]. One possible mechanism, by which GGA can exert the cytoprotection, was shown to be anti-apoptosis effect by the induction of HSP70 in renal injury model [25].

To our knowledge, however, the protective effect of GGA on drug-induced lung injury/fibrosis has not been evaluated, so we investigated whether it had a cytoprotective effect and inhibited the progression of inflammation and fibrosis in a bleomycin (BLM)-induced lung fibrosis model.

## Methods

### Reagents

Bleomycin was kindly provided by Nippon Kayaku Co., Ltd (Tokyo, Japan) and GGA was obtained from Eisai Co., Ltd (Tokyo, Japan).

### Animals

C57BL/6J (B6) mice (7- to 8-week-old females) purchased from Chubu Kagaku Shinzai Co., Ltd (Nagoya, Japan) were randomly divided into treatment groups and given access to food and water *ad libitum*. The study was conducted with the approval of Nagoya University Animal Experimentation and Ethics Committee.

### Administration of GGA in naïve mice

GGA was emulsified and prepared in 5% gum arabic and 0.6% Tween 80 for every administration to ensure a fresh suspension. Naïve mice were given a single oral dose of GGA at 600 mg/kg or vehicle. At 0, 8, 24 or 48 hours after the administration of GGA, the mice were exsanguinated by aortic perforation under pentobarbital sodium anesthesia. The lung tissues were thoroughly perfused with saline to remove blood from the vascular bed, as described before [26], and then samples were collected to evaluate the induced HSP70 expression.

Some mice were treated with the same dose of GGA at 24 hours after the first administration, and then the lung tissues were collected at 48 hours after the first dose.

### Fibrosis model

Acute pulmonary injury and fibrosis was induced by tracheal BLM administration, as described before [27]. Briefly, BLM was suspended in sterile saline and B6 mice were treated with 2 U/kg of body weight (BW) or the same volume of sterile saline (BLM mice and saline mice, respectively). The day of BLM administration was designated as day 0. To evaluate whether GGA could exert a protective effect against BLM-induced lung injury and fibrosis, we monitored BW, the change of which is assumed to closely correlate with the severity of BLM-induced lung injury [27].

### Administration of GGA in mouse fibrosis model

Mice were pretreated with single daily doses of GGA at 600 mg/kg for 7 days before BLM administration and then until the indicated time for collection of samples. They were assigned to 1) vehicle-treated BLM mice, 2) GGA-treated BLM mice, 3) vehicle-treated saline mice, and 4) GGA-treated saline mice, respectively.

### Collection of tissue specimens

On BLM days 1 and 3, lung tissues were collected from the treated mice for evaluation of HSP70 expression and TUNEL staining. On BLM day 7, some mice underwent bronchoalveolar lavage (BAL) [28]. On BLM day 14, lung tissues were collected for histological evaluation and hydroxyproline measurement.

### Western blotting analysis for HSP70 expression in vivo

Western blotting analysis was performed with minor modification [28,29]. Briefly, the resected tissues were homogenized with six volumes of ice-cold tissue lysis buffer consisting of 50 mM Tris base-pH 6.8, 2% sodium dodecyl sulfate (SDS), 150 mM NaCl, 5 mM ethylenediaminetetraacetic acid (EDTA), 1 mM phenylmethylsulfonyl fluoride (PMSF) and Complete, EDTA-free (Roche Applied Science, Penzberg, Germany). The homogenized samples were centrifuged twice for 15 minutes at 12,000 × g at 4°C, and aliquots were analyzed for total protein concentration by the Bradford assay using bovine serum albumin as the standard. Next, 10% glycerol, 0.005% bromophenol blue, and 5% β-mercaptoethanol were added to the supernatants, which were frozen until use. After correcting for the protein concentration of each sample of lung tissue, SDS-polysacrylamide electrophoresis was performed. Protein extracts at a concentration of 40 μg/lane were denatured by heating at 95°C for 4 minutes and separated on 10% SDS-PAGE gels. Electrophoresis was performed at 20 mA followed by transfer of proteins to polyvinylidene difluoride (PVDF) membranes (Millipore Corporation, Billerica, MA, USA). The transferred membranes were blocked with 5% skim milk in phosphate-buffered saline (PBS) solution (0.01 M PBS, pH 7.2) at room temperature for 1 hour and then probed with the polyclonal rabbit anti-HSP70 antibody (Clone C92F3A-5, StressGen Biotechnologies Corp., Victoria BC, Canada) (1:1000), and anti-β-actin antibody (Sigma-Aldrich, St Louis, MO, USA), diluted in the blocking solution. Membranes were subsequently rinsed three times in TBST solution (0.02 M Tris Base-pH 7.5, 0.5 M sodium chloride, and 0.1% Tween 20) for 10 minutes and exposed to the secondary HRP-conjugated anti-rabbit IgG for anti-HSP70 antibody for 1 hour at room temperature. Bound antibodies were detected with Super Signal^® ^(Pierce, Rockford, IL, USA). The blots were photographed with a CCD digital scan camera (Cool Saver, Rise & ATTO Corporation, Japan). The amount of protein on the immunoblots was quantified by image software (CS Analyzer, Rise & ATTO Corporation, Japan), and the expression levels were presented as ratios to the level of β-actin.

### Histological evaluation

On BLM day 14, lung tissue samples from treated mice were collected and fixed for 24 hours in 10% buffered formalin and then embedded in paraffin [30,31]. Sections (4 μm) were stained with hematoxylin-eosin. For the quantitative histological analysis, a numeric scale of fibrosis (Ashcroft score) was used [32]. Five sections of the entire lung stained with hematoxylin-eosin were chosen at random from each animal and more than 30 successive microscopic fields at ×100 magnification were allotted a score from 0 (normal) to 6 (severest) in a blinded fashion. In each animal the degree of cell infiltration and pneumonitis/fibrosis in the microscopic sections of lung was assessed as the mean score for the observed fields.

### Hydroxyproline analysis

The hydroxyproline content of lung tissue was measured to evaluate BLM-induced fibrosis, in which excessive collagen deposition is assumed to be one of the most representative findings [33]. After the excised lung tissue was frozen and dried, its dry weight was measured. The tissue was then hydrolyzed in 6 N HCL (10 mg/mL dry weight) at 125°C for 18 hours. Using the hydrolysis filtrate as a hydroxyproline sample, staining was performed with Ehrlich's reagent following the Prockop method, and the quantity of hydroxyproline (μg/mL) in the hydrolysis products was determined by measuring absorbance at 570 nm with a Shimadzu UV-1200 spectrophotometer.

### Immunohistochemical evaluation of HSP70 expression in vivo

To evaluate the expression of HSP70 in treated lungs, lung tissue was fixed in cold 4% paraformaldehyde solution for 6 hours, dehydrated gradually with sucrose over a period of 24 hours, fixed in OCT compound (Tissue-Tek; Miles, Elkhart, IN, USA), and frozen. Frozen lung tissue sections (4 μm) were prepared and incubated with blocking serum solution in PBS for 1 hour, followed by incubation with primary rabbit anti-HSP70 antibody (1:1000) at 4°C overnight. Antigen-antibody complexes were detected by an avidin-biotin-peroxidase technique (Vectastain ABC Kit; Vector Laboratories, Burlingame, CA, USA) [34]. Diaminobenzidine (DAB) was used to produce a brown color in the target tissue, and the sections were permanently mounted.

### TUNEL staining for tissue apoptosis in vivo

To assess the degree of lung damage, including apoptosis, in the early phase, TUNEL staining was performed using the In Situ Cell Death Detection Kit, POD (Roche Mannheim, Germany) and serial sections of the lungs collected on BLM days 1 and 3. The number of TUNEL-positive cells in the whole area of a mid-sagittal section was counted under ×400 magnification, and expressed per total cells.

### Bronchoalveolar lavage

To collect BAL fluid, the trachea was cannulated, the lungs were lavaged six times with PBS (0.5 mL each time) and approximately 2.5 mL of the instilled fluid was consistently recovered. Total cell numbers were counted with a standard hemocytometer. Cytospins were prepared by cytocentrifugation using a Cytospin 2 (Shandon Inc., Cheshire, UK) at 1,000 rpm for 5 minutes and then smears of BAL cells were stained with May-Gruenwald and Giemsa solutions. Cell differentiation was performed by counting at least 200 cells using standard hemocytologic criteria to classify them as alveolar macrophages/monocytes, neutrophils, eosinophils or lymphocytes.

### Chemokine assay

The concentration of macrophage inflammatory protein (MIP)-2 in BAL fluid was determined by ELISA [35]. All procedures were performed according to the manufacturer's instructions.

### Statistical analysis

The results were analyzed using the Mann-Whitney test for comparison between any two groups, and by nonparametric equivalents of ANOVA for multiple comparisons. P < 0.05 was considered to indicate statistical significance.

## Results

### GGA-induced HSP70 expression in the lungs in vivo

**Figure 1:**
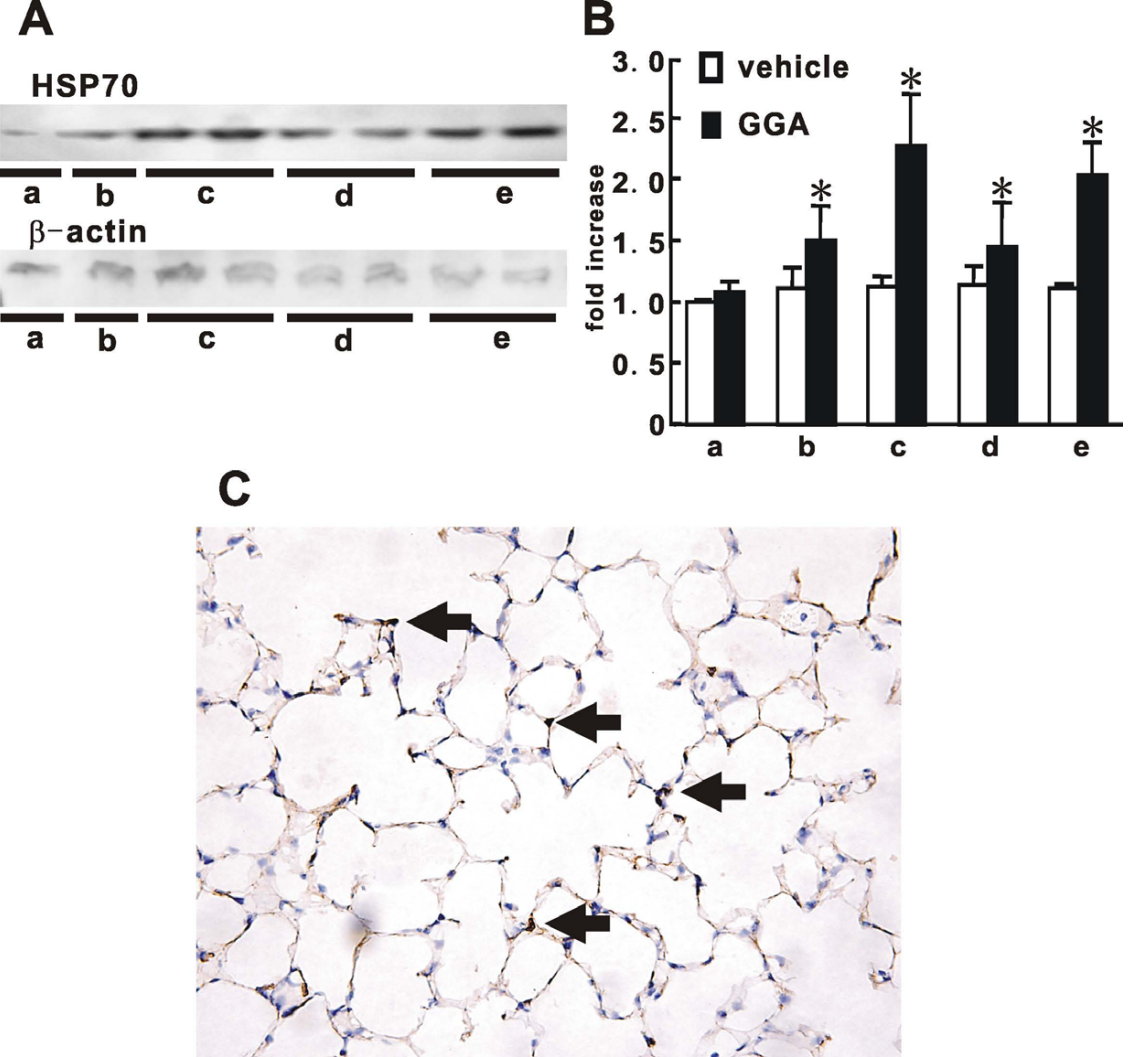
**GGA-induced expression of heat shock protein 70 (HSP70) in the lungs**. (**A**) Representative immunoblotting data for HSP70 and β-actin are shown from two similar and independent experiments. The lung tissues from 5 mice at each time were collected. (**B**) Quantitative results after normalization to the β-actin signal were summarized. Data shown represent the means ± SE. **a**; 0 hour, **b**; 8 hours, **c**; 24 hours, **d**; 48 hours, **e**; 48 hours, repeated administration of geranylgeranylacetone (GGA) at 24 hours after first administration. *Statistically significant difference (p < 0.05) in comparison with the quantitative value of HSP70 level in the lung from mice with vehicle at 0 hours. (**C**) Representative immunohistochemical staining of HSP70 in GGA-treated mice at 24 hours after GGA administration. Arrows indicate HSP70-positive cells. (× 400 magnification.)

We confirmed the induction of HSP70 by GGA and evaluated the appropriate dose and timing of GGA administration *in vivo *to induce the maximum expression of HSP70. In the naïve mice, the level of HSP70 protein in the lungs of the control vehicle-administered group was almost the same from 0 to 48 hours, and the level at 24 hours after GGA administration was two-fold greater than that at 0 hours and in the control vehicle-administered group (Fig 1A, B). The level of HSP70 protein at 48 hours, after further administration of GGA at 24 hours after first dose, was the same as that at 24 hours (Fig 1A, B: lanes c and e), whereas the level in the lungs at 48 hours after a single dose of GGA was decreased by 25% from that at 24 hours (Fig 1A, B: lanes c and d). Furthermore, we evaluated the distribution of GGA-induced HSP70 expression in the treated lung by immunohistochemical staining. Immunohistological finding indicated that steady expression of HSP70 in the lungs from GGA-treated mice appeared to be located in alveolar septa (Fig 1-C).

### Preventive effect of GGA against BLM-induced lung injury and fibrosis in vivo Monitoring of BW

**Figure 2:**
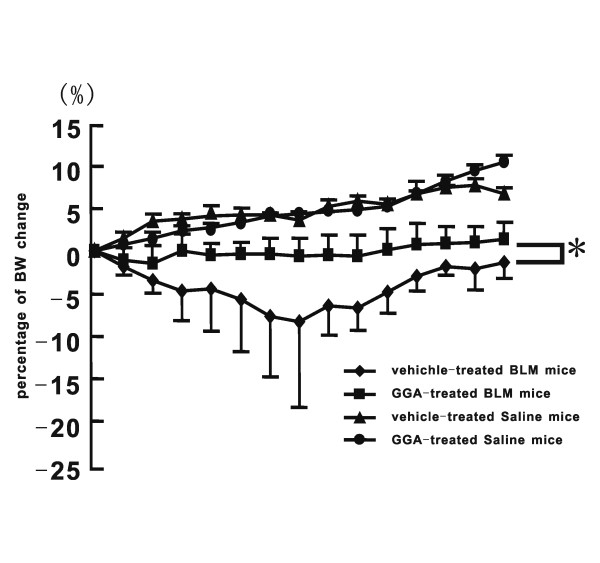
**Monitoring of change in body weight (BW) of BLM-treated mice**. Monitoring of change in body weight (BW) of BLM-treated mice at day 0 (GGA-treated mice: n = 5; GGA-treated BLM mice: n = 10; vehicle-treated BLM mice: n = 10). Data represent the mean ± SEM from two independent experiments. Comparison of the BW change in each group was analyzed using nonparametric equivalents of ANOVA. *Statistically significant difference (p < 0.05) in comparison. BLM, bleomycin; GGA, geranylgeranylacetone.

The GGA-treated mice did not show any change in BW or spontaneous movement, compared with the vehicle-treated mice (Fig 2 and data not shown). GGA-treated mice with BLM-induced lung injury (GGA-treated BLM mice) did not show substantial BW loss (1.5% decrease of BW on BLM day 2) whereas vehicle-treated mice with BLM-induced lung injury (vehicle-treated BLM mice) showed significant loss of BW (8.3% decrease on BLM day 7) (Fig 2).

### Histological evaluation of the lungs

**Figure 3:**
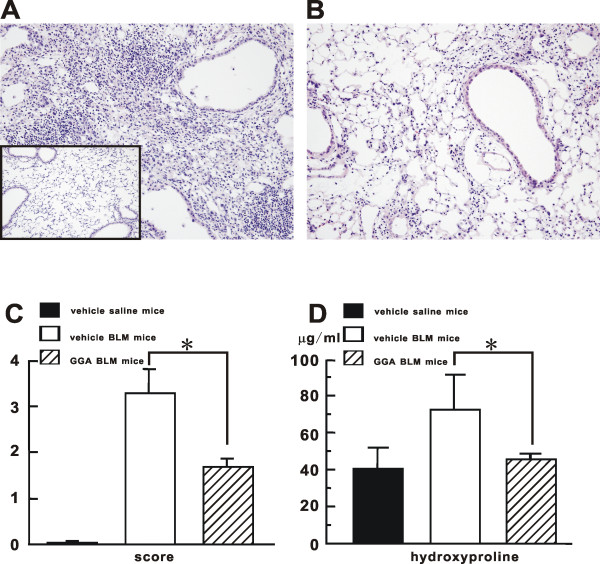
**Histological evaluation and measurement of hydroxyproline**. Histological evaluation in representative H&E stained lung sections from vehicle-treated saline mice (**Inset **in **A**), vehicle-treated BLM mice (**A**), and GGA-treated BLM mice (**B**) on BLM day14. All images at × 400 magnification. (**C**) Quantitative scoring of the severity of histological lung fibrosis (Ashcroft score). Data shown represent the mean ± SEM from two independent experiments using 5-10 mice for each group. (**D**) Hydroxyproline assay performed in the lungs from treated mice on BLM day 14. Each group had at least four mice. Data shown represent the mean ± SEM from two independent experiments. *Statistically significant difference (p < 0.05) in comparison. BLM, bleomycin; GGA, geranylgeranylacetone.

In comparison with the lungs from vehicle-treated saline mice (inset in Fig 3A), the lungs of vehicle-treated BLM mice revealed severe inflammatory cell infiltration in the subepithelial and subpleural regions with prominent disorganized thickening of the alveolar septa, resulting in the loss of normal structure (Fig 3A). GGA-treated BLM mice showed a significant decrease in both interstitial infiltration and airspace cellularity and there were less histological changes, compared with vehicle-treated BLM mice (Fig 3B). Quantitative histological scoring of lung injury showed that the score in the vehicle-treated BLM mice was significantly higher (3.3 ± 0.5) than that in the GGA-treated BLM mice (1.7 ± 0.2) (Fig 3C). As the collagen content of the lung, as measured by hydroxyproline content, it was also significantly lower in GGA-treated BLM mice as compared with vehicle-treated BLM mice (vehicle-treated BLM mice: 72.5 ± 18.8 μg/mL; GGA-treated BLM mice: 45.3 ± 3.5 μg/mL) (Fig 3D).

### Cellular characterization and MIP-2 level in BAL fluid

**Figure 4:**
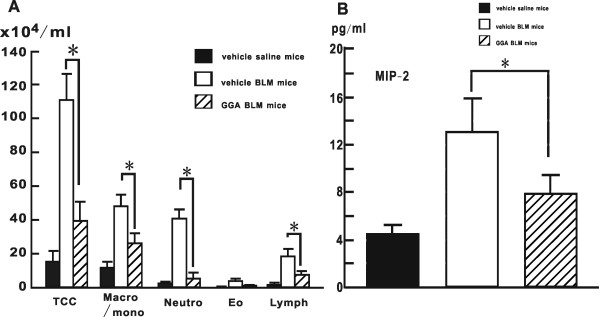
**Cellular profiles and macrophage inflammatory protein-2 (MIP-2) level in bronchoalveolar lavage (BAL) fluid**. (**A**) Total cell counts and cell differentiation in BAL fluid from treated mice on BLM day 7. At least four mice were prepared for each group in each experiment. Data shown represent the mean ± SEM from two independent experiments. TCC: total cell counts; Macro/mono: alveolar macrophages/monocytes; Neutro: neutrophils; Eo: eosinophils; Lym: lymphocytes. (**B**) MIP-2 levels in BAL fluid from treated mice on BLM day 7 was evaluated by ELISA assay. At least four mice were prepared for each group in each experiment. Data shown represent the mean ± SEM from two independent experiments. *Statistically significant difference (p < 0.05) in comparison.

As previously reported, analysis of the cellular profile in BAL fluid supports the histological findings [32]. The total cell number in the BAL fluid increased significantly in vehicle-treated BLM mice, and was clearly inhibited in GGA-treated BLM mice (vehicle-treated BLM mice: 110.3 ± 15.7 × 10^4^/mL; GGA-treated BLM mice: 39.3 ± 11.1 × 10^4^/mL) (Fig 4A). GGA treatment significantly suppressed the number of neutrophils in BAL fluid, as well as alveolar macrophages/monocytes (neutrophils: 40.5 ± 5.4 × 10^4^/mL in vehicle-treated BLM mice and 5.2 ± 3.3 × 10^4^/mL in GGA-treated BLM mice) (Fig 4A). MIP-2 levels in BAL fluid from GGA-treated BLM mice were significantly suppressed compared with those from vehicle-treated BLM mice (vehicle-treated BLM mice; 13.0 ± 2.8 pg/mL, GGA-treated BLM mice; 7.8 ± 1.6/mL, respectively) (Fig 4-B).

### Cytoprotective effect of GGA in BLM-induced lung injury by TUNEL assay

**Figure 5:**
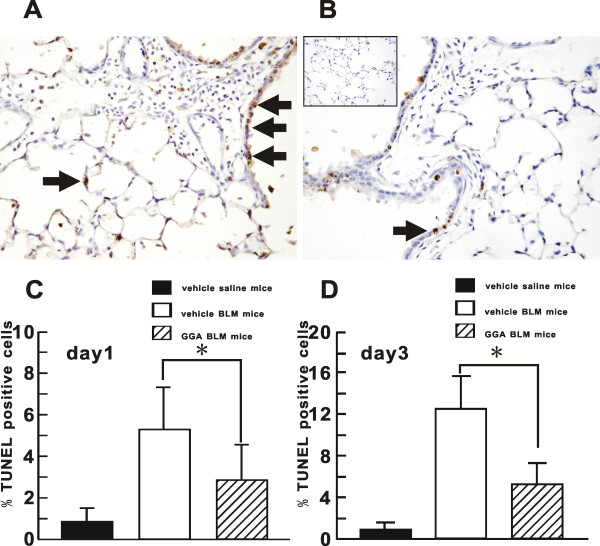
**Evaluation of cytoprotective effect of GGA against lung injury by *in vivo *TUNEL assay**. Evaluation of cytoprotective effect of GGA against lung injury by *in vivo *TUNEL assay of the lungs from treated mice on BLM day 1 and day 3. Representative lung sections from day3 vehicle-treated BLM mice (**A**) and GGA-treated BLM mice (**B**) were shown. TUNEL-positive cells (**arrows **in **A **and **B**) were counted in the whole area of the mid-sagittal section (day 1 in **C **and day 3 in **D**), expressed per total cell counts. At least five mice were prepared for each group in each experiment. Data shown represent the mean ± SEM from two independent experiments. *Statistically significant difference (p < 0.05) in comparison. BLM, bleomycin; GGA, geranylgeranylacetone.

Until day 3, the histological accumulation of inflammatory cells was still limited and the normal structure of lung remained (Fig 5C, D), but by day 14 large number of inflammatory cells had accumulated and the normal structure was severely damaged in vehicle-treated BLM mice (Fig 3A). As shown in Fig 5, mainly epithelial cells were TUNEL-positive, although some cells beneath the bronchi, most likely inflammatory cells and mesenchymal cells, were also TUNEL-positive. The ratio (%) of TUNEL-positive cells in the lungs from vehicle-treated BLM mice on BLM days 1 and 3 was significantly larger that those from GGA-treated BLM mice at day 1 and day 3 (BLM day 1: 5.0 ± 1.9% in vehicle-treated BLM mice and 2.6 ± 1.5% in GGA-treated BLM mice; BLM day 3: 12.3 ± 3.1% in vehicle-treated BLM mice and 4.7 ± 1.8% in GGA-treated BLM mice) (Fig 5C, D).

### In vivo GGA-induced HSP70 protein expression in BLM-induced lung injury model

**Figure 6:**
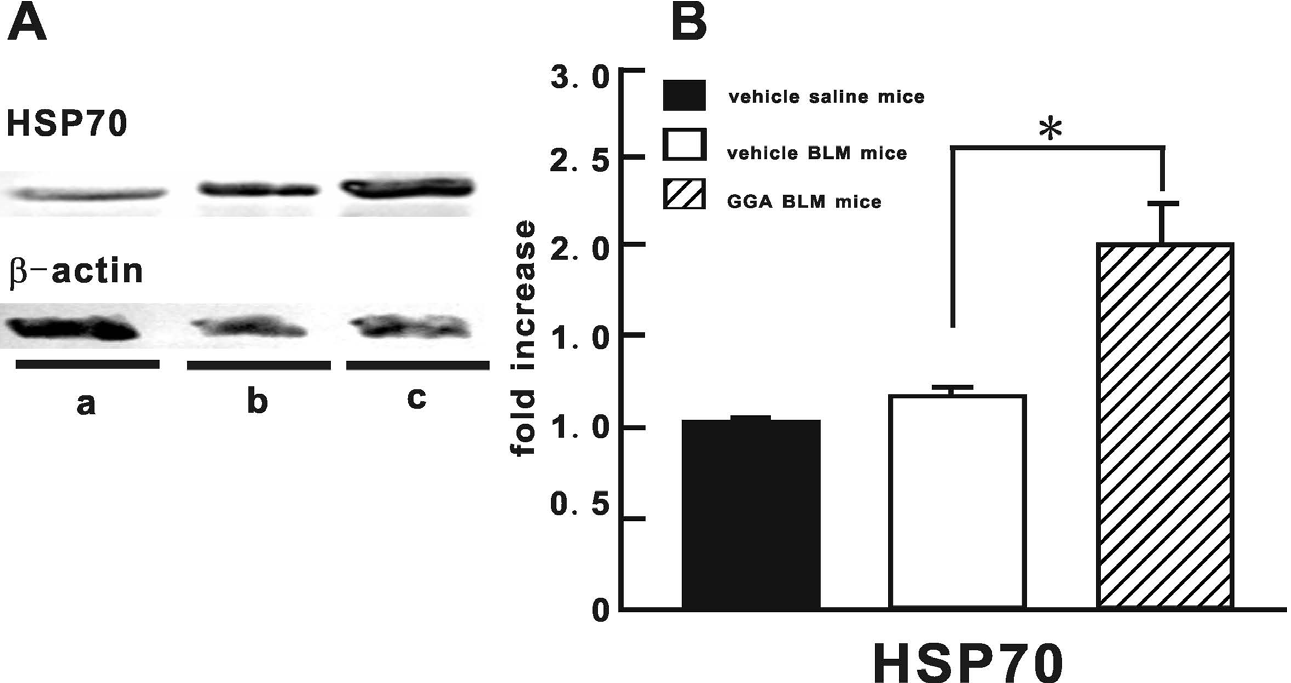
**HSP70 protein expression *in vivo *in BLM-induced lung injury model**. (**A**) Representative immunoblotting data for HSP70 and β-actin on day 3 from two similar and independent experiments. **a**; vehicle saline mice, **b**; vehicle BLM mice, **c**; GGA BLM mice. (**B**) Quantitative results of HSP70 after normalization to β-actin signal were summarized. At least five mice were prepared for each group from two independent experiments. Data shown represent the mean ± SEM from two independent experiments. *Statistically significant difference (p < 0.05) in comparison. BLM, bleomycin; GGA, geranylgeranylacetone; HSP70, heat shock protein 70.

Western blotting analysis showed that the level of HSP70 protein expression in the lungs from GGA-treated BLM mice on BLM day 3 was significantly greater than in vehicle-treated BLM mice at the same time point (vehicle-treated BLM mice: 1.1 ± 0.1; GGA-treated BLM mice: 1.9 ± 0.2) and that the levels in vehicle-treated saline mice were the same as those in vehicle-treated BLM mice (Fig 6).

## Discussion

We have demonstrated for the first time that repeated oral administration of GGA exerted a protective effect against lung injury in a drug-induced lung injury/fibrosis animal model and subsequently had a suppressive effect on the fibrotic reaction, related to the induction of HSP70, without remarkable adverse events.

While there is no completely satisfactory animal model of IIPs and drug-induced interstitial pneumonitis, the intra-tracheal BLM-induced lung injury model is relatively well-characterized and does exhibit certain features found in the human disease. There are clear limitations to this model in terms of its self-limiting nature, the rapidity of its development and close association with inflammation that accompanies the lung injury [26,33]. However, the model is useful for identification of mechanisms and pathogenetic clues that may be relevant to the human disease. Finding such clues may be helpful in providing the basis for human studies to confirm their relevance.

Our finding that BLM-treated mice lost BW, most likely because of lung injury/fibrosis, was compatible with the change in BW observed in a previous study [27]. Furthermore, we confirmed in detail that BLM induced the following sequential events as follows: apoptosis, accumulation of inflammatory cells, increased chemokine (MIP-2) in BAL fluid, and fibrosis. As it has been accumulated the finding that AECs and endothelial cells injury and death are a critical finding in both IIPs and BLM-induced lung fibrosis model [13,36], apoptosis is assumed to be one of the most important mechanisms, by which fibrotic reaction was mediated. Lung constituents such as AECs and endothelial cells are assumed to be substantial targets in BLM-induced apoptosis *in vivo *[13,36]. Many studies suggest that AEC injury and apoptosis, consistent findings not only in human IPF/UIP but also in BLM-induced lung fibrosis model, might induce initial recruitment of leukocytes, partly because of cytokines and chemokines released from damaged tissues [13,37-40]. In particular, MIP-2 could be the most specific chemokine for the recruitment of neutrophils, by which lung injury might be amplified and developed [28]. Therefore, it has been assumed that intra-tracheal administration of BLM could cause lung injury, partly through the increasing MIP-2 production in the injured lungs, triggered by AEC damage/apoptosis [26].

In our present study, the inflammatory process in the BLM-induced lung injury/fibrosis model was inhibited in the GGA treated-BLM mice, compared with vehicle treated-BLM mice, resulting in the attenuation of the degree of lung fibrosis. These findings were supported by BAL fluid analyses, including inflammatory cell counts and quantification of MIP-2 between GGA treated-BLM mice and vehicle treated-BLM mice. Indeed, the sequential fibrosis was significantly attenuated, assessed by the Ashcroft score and the hydroxyproline content of lung tissue. Although the intra-tracheal BLM induced lung fibrosis model might not be a completely satisfactory animal model for drug-induced lung injury/fibrosis, these findings, in which the preventive effect of GGA against BLM-induced lung fibrosis was successfully shown, could be the clinically helpful clue in providing the basis on the therapeutic strategy for drug-induce lung injury/fibrosis.

Importantly, oral treatment with GGA did not cause any adverse effects and the mice exhibited normal BW gain and activity during the experiment. GGA treatment significantly ameliorated BW loss in BLM-treated mice during the experiment, suggesting that GGA treatment could exert a general protective effect on damage caused by BLM administration.

Several reports have suggested that protective effect of GGA might be, in part, due to inhibit apoptosis process through induction of HSP70 [25,41-43]. Therefore, we evaluated the expression of HSP70 *in vivo *to illuminate the possible molecular mechanism, by which administration of GGA could prevent BLM-induced lung injury/fibrosis *in vivo*.

Changes in the expression of HSP70 with time after oral administration of 200 mg/kg of GGA in the rat kidney [25] and heart [41] have been reported. While one study reported that induction of HSP70 at 24 hours after a single oral administration of GGA at 200 mg/kg was not detected in rat lungs [44], the other report showed the induction of HSP70 in the rat lungs by intra-arterial administration of 1000 mg/kg of GGA [45]. To our knowledge, there has not been a report demonstrating the induction of HSP70 by oral administration of GGA. In our study, oral administration of GGA at 600 mg/kg clearly induced increased expression of HSP70 in mouse lungs beginning at 8 hours and reaching maximal levels by 24 hours, in comparison with the faint expression of HSP70 before treatment with GGA, although HSP70 expression at 48 hours after a single dose of GGA reverted back to the same level as at 8 hours. According to these results, sequential and repeated oral-administration of GGA before and after the administration of BLM should be needed to achieve a sufficient protective effect against BLM-induced lung injury and fibrosis.

To determine whether anti-inflammatory effect by GGA *in vivo *may be due to the anti-apoptosis effect by the attribution of the *de novo *induction of HSP70 or not, the expression of HSP70 in the treated lungs were performed by western blotting. The slight, but not significant increasing HSP70 expression in the lungs from vehicle treated BLM mice was observed in our present study, compared with those from vehicle treated BLM mice. It is suggested in our present study that the induction of HSP70 by BLM was limited, although it was reported that BLM induced the HSP70 promoter in cultured cells [46] and that the modulation of HSP70 expression by BLM might vary on the cell types such as fibroblasts and alveolar macrophages [47,48]. On the other hand, GGA administration induced the significantly increasing expression of HSP70 in BLM-treated lungs, compared with that of BLM-treated lung with vehicle. To evaluate whether GGA treatment can prevent the apoptosis in BLM-treated lungs, TUNEL staining was performed for the lungs at BLM day1 and day3. TUNEL staining showed that TUNEL-positive cells were observed mainly in epithelial cells of BLM-treated lungs at day1 and day3. The number of TUNEL-positive cells in the lungs from GGA-treated BLM mice was significantly smaller than those from vehicle-treated mice with vehicle. Furthermore, previous study using quercetin, an inhibitor to the induction of HSPs, showed that quercetin suppressed the expression of HSP70 and abolished the cytoprotective effect of GGA in renal injury model [25]. Taken together with the distribution of steady expression of HSP70 in alveolar septa of the lungs from GGA-treated mice, our findings allow us to speculate that the *in vivo *induction of HSP70 in this model by pretreatment of GGA might partly protect against BLM-induced cell damage and apoptosis, possibly in AECs, resulting in prevention of sequential inflammation and fibrosis. Although the up-regulation of HSP70 have been shown in human tissue injury [24], the HSP70 expression has not yet been determined in human lung injury. In this study, we focused on the early effect of GGA on HSP70 induction in BLM-induced lung injury and fibrosis model *in vivo *since it is well assumed that the appropriate control for the initial triggers on lung injury/fibrosis may result in the attenuation of lung injury/fibrosis. Further investigations 1) for the contribution of GGA on HSP70 induction at later phase in this model, 2) for the preventive effect of GGA against the established lung fibrosis, and if so, 3) for human lung injury, are warranted. Although it is also well known that both methotrexate and BLM show the cytotoxic effect for lung constituents, resulting in apoptosis [49,50], they are assumed to show different heat shock responses [47]. It might need to be elucidated by further study whether GGA might also protect lung injury due to methotrexate or not.

## Conclusion

In conclusion, our present findings are the first to show that pre-treatment of GGA or GGA-induced derivatives may give us a new strategy to prevent RA patients from interstitial pneumonitis induced by DMARDs after due consideration of both the protocol design *in vivo *with the higher experimental dose of GGA than the clinical dose and the known limitations of BLM-induced fibrosis.

## List of Abbreviations

AECs: alveolar epithelial cells; ANOVA: analysis of variance; BAL: bronchoalveolar lavage; BLM: bleomycin; BW: body weight; DAB: diaminobenzidine; DMARDs: disease-modifying anti-rheumatic drugs; EDTA: ethylenediaminetetraacetic acid; ELISA: enzyme-linked immunosorbent assay; GGA: Geranylgeranylacetone; HSP: heat shock protein; IIP: idiopathic interstitial pneumonitis; MIP-2: macrophage inflammatory protein-2; PBS: phosphate-buffered saline; PMSF: phenylmethylsulfonyl fluoride; PVDF: polyvinylidene difluoride; RA: rheumatoid arthritis; SDS: sodium dodecyl sulfate; TUNEL: TdT-mediated dUTP nick and labeling.

## Competing interests

The authors declare that they have no competing interests.

## Authors' contributions

Authors TF and NH contributed equally to this work. Authors TF, NH, MJ, YH, TK and NI were all involved in the design and implementation of the study including data collection and measurement. Authors TF, NH, MJ, and TK were involved in the analysis and interpretation of the data. All authors read and approved the final manuscript.

## Pre-publication history

The pre-publication history for this paper can be accessed here:


